# Boric acid transport activity of marine teleost aquaporins expressed in *Xenopus* oocytes

**DOI:** 10.14814/phy2.15655

**Published:** 2023-03-26

**Authors:** Shiori Kumagai, Erika Watanabe, Naoko Hayashi, Yuuri Kimura, Takehiro Kamiya, Ayumi Nagashima, Kazutaka Ushio, Genki Imaizumi, Juhyun Kim, Keijiro Munakata, Takahiro Umezawa, Shigehisa Hirose, Koji Kasai, Toru Fujiwara, Michael F. Romero, Akira Kato

**Affiliations:** ^1^ School of Life Science and Technology Tokyo Institute of Technology Yokohama Japan; ^2^ Graduate School of Bioscience and Biotechnology Tokyo Institute of Technology Yokohama Japan; ^3^ Department of Applied Biological Chemistry, Graduate School of Agricultural and Life Sciences The University of Tokyo Tokyo Japan; ^4^ Department of Physiology and Biomedical Engineering Mayo Clinic College of Medicine Rochester Minnesota United States; ^5^ Nephrology & Hypertension Mayo Clinic College of Medicine Rochester Minnesota United States; ^6^ O'Brien Urology Research Center Mayo Clinic College of Medicine Rochester Minnesota United States; ^7^ Center for Biological Resources and Informatics Tokyo Institute of Technology Yokohama Japan

**Keywords:** aquaglyceroporin, aquaporin, boric acid, electrophysiology, marine teleost

## Abstract

Marine teleosts ingest large amounts of seawater containing various ions, including 0.4 mM boric acid, which can accumulate at toxic levels in the body. However, the molecular mechanisms by which marine teleosts absorb and excrete boric acid are not well understood. Aquaporins (Aqps) are homologous to the nodulin‐like intrinsic protein (NIP) family of plant boric acid channels. To investigate the potential roles of Aqps on boric acid transport across the plasma membrane in marine teleosts, we analyzed the function of Aqps of Japanese pufferfish (*Takifugu rubripes*) expressed in *Xenopus laevis* oocytes. *Takifugu* genome database contains 16 genes encoding the aquaporin family members (*aqp0a*, *aqp0b*, *aqp1aa*, *aqp1ab*, *aqp3a*, *aqp4a*, *aqp7*, *aqp8bb*, *aqp9a*, *aqp9b*, *aqp10aa*, *aqp10bb*, *aqp11a*, *aqp11b*, *aqp12*, and *aqp14*). When *T. rubripes* Aqps (TrAqps) were expressed in *X. laevis* oocytes, a swelling assay showed that boric acid permeability was significantly increased in oocytes expressing TrAqp3a, 7, 8bb, 9a, and 9b. The influx of boric acid into these oocytes was also confirmed by elemental quantification. Electrophysiological analysis using a pH microelectrode showed that these TrAqps increase B(OH)_3_ permeability. These results indicate that TrAqp3a, 7, 8bb, 9a, and 9b act as boric acid transport systems, likely as channels, in marine teleosts.

## Graphical abstract

Boric acid permeability of oocytes expressing pufferfish Aqp3a, 7, 8bb, 9a, and 9b. Representative traces of changes in the intracellular pH and membrane potential of an oocyte expressing pufferfish Aqps are shown. 10BA, 10 mM boric acid.

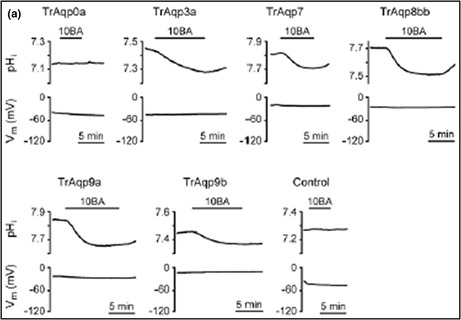

## 1 INTRODUCTION

As a micronutrient, boron is essential for a wide range of organisms, including plants, animals, fungi, and bacteria (Miwa & Fujiwara, 2010; Nielsen, 2014; Uluisik et al., 2018). In higher plants, boron is essential as a component of the cell wall matrix, and its deficiency affects the growth and expansion of plant organs (Blevins & Lukaszewski, 1998; Tanaka & Fujiwara, 2008). Boron deficiency impairs embryogenesis in aquatic vertebrate animals such as *Xenopus laevis* (Fort et al., 2002) and zebrafish (Rowe & Eckhert, 1999). In mammals, boron provides many beneficial health effects and is considered nutritionally important (Nielsen, 2014; Uluisik et al., 2018). Boron plays beneficial roles in bone growth and maintenance, hormone functions, brain function, amelioration of arthritis, and cancer risk reduction (Nielsen, 2014, Uluisik et al., 2018). Boric acid is rapidly absorbed from the intestine and excreted in the urine (Uluisik et al., 2018). In higher plants, factors involved in boron homeostasis have been identified (Miwa & Fujiwara, 2010; Onuh & Miwa, 2021; Yoshinari & Takano, 2017). However, the biochemical mechanisms of action of boron and molecules that maintain boron homeostasis are poorly understood in vertebrate animals.

Excess boron or boric acid intake is toxic to a wide range of organisms. In plants, boron toxicity reduces the growth and expansion of plant organs, such as leaves, roots, flowers, fruits, and seeds (Miwa & Fujiwara, 2010). For adult humans, an acceptable safe range of boron intake is 1.0–13 mg boron/day (Nielsen, 1996). Gastrointestinal, cardiovascular, hepatic, renal, and central nervous system effects, dermatitis, erythema, and death have been observed in children and adults exposed to ≥84 mg boron/kg (Khaliq et al., 2018; Uluisik et al., 2018). The reported acute oral LD_50_ values in mice and rats are 600 and 460–900 mg boron/kg body weight, respectively (Pfeiffer et al., 1945; Weir Jr. & Fisher, 1972). The reported fish LC_50_ values under various test conditions are highly variable ranging from 3.63 to 1000 mg boron/L (Schoderboeck et al., 2011). The LC_50_ (16‐day) value of sockeye salmon (*Oncorhynchus nerka*) in seawater (SW) is 12 mg boron/L (Eisler, 1990). Concentrations of 100–300 mg boron/L can cause the death of various aquatic vertebrates (Birge & Black, 1977). The lowest observed effect concentrations (LOECs) for rainbow trout (*Oncorhynchus mykiss*) are 0.10 mg/L. Exposure of juvenile rainbow trout to freshwater (FW) containing 17 or 170 mg boron/L causes acute histopathological alterations in the gill and kidney (Topal et al., 2016), and the acceptable level of boron exposure to rainbow trout was concluded as ≤1.0 mg boron/L (Loewengart, 2001). Feeding rainbow trout with 0.05% boron‐supplemented feed stimulates the growth parameters, but 0.20% of boron supplementation inhibits the growth and causes degenerative and necrotic changes in the liver, gill, kidney, skeletal muscle, spleen, and brain (Öz et al., 2018; Öz et al., 2020).

The plasma of marine teleosts has an ionic composition and osmolarity similar to that of mammals and FW teleosts, and is hypotonic to seawater. To avoid dehydration, marine fish ingest large amounts of SW, absorb water and electrolytes in the intestine, and then excrete excess electrolytes back into the SW via the gills (branchial chloride cells) (e.g., Na^+^ and Cl^−^) or into the iso‐osmotic urine via the kidneys (e.g., Mg^2+^ and SO_4_^2−^) (Marshall & Grosell, 2006). In seawater, the predominant boron species is boric acid (Thompson et al., 1976), and borate or boric acid occurs naturally at 4.5–5.5 mg boron/L (0.4–0.5 mM) (Eisler, 1990). Therefore, SW fishes are exposed to a continuous influx of boric acid, which must be excreted. Recently, we analyzed boric acid and boron concentrations in the body fluids of the river pufferfish (*Takifugu obscurus*, euryhaline) and the Japanese pufferfish (*Takifugu rubripes*, marine) acclimated in FW, brackish water (BW), and SW (Kato et al., 2022). In SW, pufferfishes maintained serum boric acid concentration at 0.02–0.06 mM (the boric acid concentration of the intestinal fluid was 0.04–0.15 mM). Interestingly, the concentration of boric acid in the bladder urine of SW‐acclimated pufferfishes was 14–19 mM, which was 150–950‐fold greater than that of FW‐ or BW‐acclimated pufferfishes. These results indicate that marine teleosts concentrate boric acid into the iso‐osmotic urine, which is then excreted.

Two boron transport systems have been initially identified in plants, that is, active transport by the BOR transporter and facilitated transport by nodulin‐like intrinsic protein (NIP) channels (Miwa & Fujiwara, 2010; Tanaka & Fujiwara, 2008). BOR1, which was initially identified in *Arabidopsis*, is homologous to members of the mammalian solute carrier 4 (SLC4), family of bicarbonate transporters. The human homolog of BOR1 is SLC4A11 (Parker et al., 2001), which was initially characterized as a Na^+^‐coupled borate cotransporter NaBC1 (Park et al., 2004); however, recent studies have shown that mammalian SLC4A11 enables H^+^/OH^−^ transport in both Na^+^‐independent and Na^+^‐coupled modes but does not transport borate (Kao et al., 2020; Loganathan et al., 2016; Myers et al., 2016; Ogando et al., 2013). In contrast, recent functional analysis of Slc4a11A, a paralog of SLC4A11 in pufferfishes, showed that it functions as an electrogenic boric acid transporter (Kato et al., 2022).

The NIP family is another family of boron transporters that belong to the water channel superfamily. NIP5;1 was initially identified as a boric acid channel required for boric acid uptake and normal growth in *Arabidopsis* (Takano et al., 2006). The Aqp family consists of 13 members in humans (Agre et al., 2002; Azad et al., 2021; Borgnia et al., 1999) and 18 members in zebrafish (Tingaud‐Sequeira et al., 2010). Detailed analyses of Aqp superfamily of various vertebrate species clarified that vertebrate aquaporins are distributed in 17 subfamilies (Aqp0‐16) and belong to 4 subfamilies, that is, orthodox or classical Aqps that selectively transport water; aquaglyceroporins that transport water and other small neutral solutes, such as glycerol and urea; Aqp8‐type aquaporins; and unorthodox or super Aqps, whose function is still uncertain (Chauvigne et al., 2019; Finn et al., 2014; Finn & Cerda, 2015; Yilmaz et al., 2020). In humans (*Homo sapiens*), HsAqp3, 7, 9, and 10 are recognized as aquaglyceroporins. In zebrafish (*Danio rerio*), DrAqp3, 7, 9, and 10 are recognized as aquaglyceroporins, and DrAqp8 is recognized as a water and urea channel (Tingaud‐Sequeira et al., 2010). Recently, we analyzed the boric acid transport activity of human Aqps expressed in *Xenopus laevis* oocytes and showed that human aquaglyceroporins (HsAqp3, 7, 9, and 10) and HsAqp8 act as boric acid transport systems, likely as channels (Ushio et al., 2022). To determine whether Aqps act as boric acid channels in marine teleosts, we expressed pufferfish (*T*. *rubripes*) Aqps in oocytes and analyzed their activity through swelling assays, elemental quantification, and electrophysiology. The results indicated that TrAqp3a, 7, 8bb, 9a, and 9b act as boric acid transport systems in marine teleosts.

## 2 MATERIALS AND METHODS

### 2.1 Database search and phylogenetic analysis

To identify Aqp genes in the Japanese pufferfish (*Takifugu rubripes*), BLAST searches were performed on the *Takifugu rubripes* genomic database in Ensemble Genome Browser (http://www.ensembl.org/index.html). To analyze the phylogenetic relationships of Aqps in *Takifugu rubripes* and other related animals, amino acid sequences of Aqps were obtained from genome databases of human (*Homo sapiens*), mouse (*Mus musculus*), western clawed frog (*Xenopus tropicalis*), spotted green pufferfish (*Tetraodon nigroviridis*), and zebrafish (*Danio rerio*) in Ensemble Genome Browser (Table S1). The amino acid sequences were aligned using ClustalW software (Thompson et al., 1994) and a phylogenetic tree was constructed using MEGA software (Kumar et al., 2016) based on the maximum‐likelihood (ML) method with 100 bootstrap replications.

### 2.2 Semi‐quantitative reverse transcriptase‐polymerase chain reaction (RT‐PCR)

Semi‐quantitative RT‐PCR was performed using total RNA isolated from the *Takifugu rubripes* tissues (Nag et al., 2006; Nakada et al., 2007; Tran et al., 2006). First‐strand complementary DNA was synthesized from 5 μg total RNA using the SuperScript III First‐Strand Synthesis System (Thermo Fisher Scientific) with Oligo(dT) primers and analyzed by RT‐PCR as described previously (Tran et al., 2006). The cDNA was diluted eight times with nuclease‐free water and used as the template for PCR with gene‐specific primers (Table 1). Each reaction mixture (final volume, 12.5 μL) consisted of 0.25 μL cDNA (template), primers (individual final concentration, 0.25 μM), 6.25 μL GoTaq Green Master Mix (2×; Promega). The PCR conditions were as follows: initial denaturation at 94°C for 2 min, followed by 28 or 33 cycles at 94°C for 15 s (denaturation), 52 (*aqp10aa* and *aqp10bb*) or 54°C (the others) for 30 s (annealing), 72°C for 1 min (extension), and a final extension at 72°C for 7 min. After amplification, the PCR mixture was diluted at 1:10 (*aqp10aa*, *aqp10bb*, *aqp11a*, and *aqp11b*), 1:20 (*aqp0a*, *aqp0b*, *aqp3a*, *aqp4a*, *aqp7*, *aqp8bb*, *aqp9a*, *aqp9b*, *aqp12*, and *aqp14*), or 1:160 (*aqp1aa*, *aqp1ab,* and *actb*) and was loaded 3 μL each onto a microchip electrophoresis system for DNA/RNA analysis MCE‐202 MultiNA (Shimadzu) using a DNA‐12000 reagent kit (Shimadzu) according to the manufacturer's instructions. Electrophoresis results were analyzed using the MultiNA Viewer software (Shimadzu).

**TABLE 1:** List of primers used for the polymerase chain reaction amplification of *aqp* gene family in *Takifugu rubripes*.

Gene	Accession	Remarks	Direction	Sequence (5′ to 3′)
*aqp0a*	LC735281, XM_003963091, ENSTRUT00000009841	RT‐PCR	Forward	TTACATTTGCCTACCTGATTGGCTC
			Reverse	GAACAGCATGAAGTCGTATAGCAGAG
		cDNA cloning	Forward	ATGTGGGAGTTCAGGTCCATGTCTTTCTGG
			Reverse	TTTATAGGGCCTGTGTCTTTAGCTCGATGG
*aqp0b*	LC735282, XM_003963426, ENSTRUT00000040237	RT‐PCR	Forward	ATTTTCTACATGGCCGCCCAGTGTC
			Reverse	CGTGGGAAGAGCATGAAATCGTACAGG
		cDNA cloning	Forward	ATGTGGGAGTTCCGTTCCATGAATTTTTGG
			Reverse	TTTATAGGGTCTGCGTCTTGAGCTCGATGG
*aqp1aa*	LC735283, XM_003975326, ENSTRUT00000083310	RT‐PCR	Forward	CTATTGGGAACAAGAACAACAGCAA
			Reverse	GTGAAATCGTTCAGGATCAAAGCG
		cDNA cloning	Forward	ATGAGAGAGTTGAAGAGCAAGGACTTCTGG
			Reverse	TCTATTTTGATGTCATCTCCACTGTGGTGT
*aqp1ab*	LC735284, XM_003975371, ENSTRUT00000034800.3	RT‐PCR	Forward	TTGCTCTTCATATTCTTCGCTCTGTG
			Reverse	CACATCCGGTGAAACTAATTGCTG
		cDNA cloning	Forward	ATGGCAGAGATAAAGCGCTGGACGTTTTGG
			Reverse	TTCACTGTTTTGGCCATTGACTTGGCCCCG
*aqp3a*	LC735285, XM_003975233	RT‐PCR	Forward	GCACTCATAGTTTGTATTCTGGCTATC
			Reverse	TCACAGGATGTCTTTGGTGTGTTTG
		cDNA cloning	Forward	ccaccATGGGCAGACAGAAGGTGTA
			Reverse	cagcagccTCACAGGATGTCTTTGG
*aqp4a*	LC735286, XM_011618835, ENSTRUT00000076888	RT‐PCR	Forward	CTTTCAAAGGCATCTGGACCAAG
			Reverse	ATCTGAGAGTTCACCTCCGTGAC
		cDNA cloning	Forward	ctgacatcATGGCGGCTTTCAAAGG
			Reverse	ccgcacgtgatagTCATACGGAGGA
*aqp7*	LC735287, XM_003973563, ENSTRUT00000039663	RT‐PCR	Forward	GAAGGAAATGGCAGAATCTATGGAAC
			Reverse	CAGTTCCACCATGATCTTGTAAAGC
		cDNA cloning	Forward	ccaccATGGAACTGGGCGTCTCTCA
			Reverse	cgggggacTCACACACAAACATTAT
*aqp8bb*	LC735288, XM_003964545, ENSTRUT00000007374	RT‐PCR	Forward	ATGAACATGGTGTTCCCGTATCTC
			Reverse	CAAAGAAAGCACCATCGTATCAGAAC
		cDNA cloning	Forward	ccaccATGGCGGACGAGAAGATGGA
			Reverse	ctcttccacttcgccTCACTTCAGA
*aqp9a*	LC735289, XM_003967709, ENSTRUT00000021115	RT‐PCR	Forward	ATTCTGGGTAAGCTGAAGATCTGGAA
			Reverse	CTCATGGTGATCATCTCGTATTTGTC
		cDNA cloning	Forward	cATGAGGAAACGCTGTGCCATCAAA
			Reverse	ctTCAGCTCATGGTGATCATCTCGT
*aqp9b*	LC735290, XM_003969979, ENSTRUT00000045810	RT‐PCR	Forward	AAAGGTATGGAGCCTCTGTGC
			Reverse	CCTCATTAAACTCTGGATTCGCTATCA
		cDNA cloning	Forward	ATGGAAAGCGAGAGCAAGAGGAAGATGA
			Reverse	TTAGGTCATGGTCATGATTTCATATTTGT
*aqp10aa*	LC735291, XM_011605829, ENSTRUT00000066901	RT‐PCR	Forward	CATGCTGATGCTGTGCATTCTG
			Reverse	TGGATCTGGGAGGTGCAAAGAA
		cDNA cloning	Forward	ATGAAGGCTTTGAAAACCAGGAACGCTCTG
			Reverse	TTCAAACCAGTACTCCTGGTGTCTCTCGAA
*aqp10bb*	LC735292, XM_003969282, ENSTRUT00000024481	RT‐PCR	Forward	CTGAAGAACTGTCGGATCAAGAAC
			Reverse	GTAGGTCTGGATGGCATCATAATA
		cDNA cloning	Forward	ATGGACGGGCTCCTGAAGAACTGTCGGATC
			Reverse	TTCACTGAGCTTTTCCATCTCGCTCCAGCT
*aqp11a*	LC735293, XM_003968221, ENSTRUT00000017753	RT‐PCR	Forward	TCTGAAGGAGAAATACCGCGTACA
			Reverse	ATCTTGGTCTAAACTCCTGTTTCTCAC
		cDNA cloning	Forward	ATGGCCGCAGACGTGCTCGTATCGCTGGGG
			Reverse	TTCATGTCATCTTCTTCTTCTTCATTGAAG
*aqp11b*	LC735294, XM_003970961, ENSTRUT00000050959	RT‐PCR	Forward	GAGTTGCCCGAAGTTTCCTG
			Reverse	ATAGATGAAGCAGTACTCCAGGTG
		cDNA cloning	Forward	ATGAGCGACTTGCGCGTGTCAGTGGCGGTG
			Reverse	TCTAGGCCAACTTGTGCTTGTGGCTCTTTC
*aqp12*	LC735295, XM_003973857, ENSTRUT00000041698	RT‐PCR	Forward	ATCTCTAGGATATTTCCTGGGTGGA
			Reverse	AAATAATCCCAGGTTAGATTGGGTTGG
		cDNA cloning	Forward	ccaccATGTCCGGACTGAACGCATC
			Reverse	ctttcttTCACATTTTCTTCTTTTC
*aqp14*	LC735296, XM_003963431, ENSTRUT00000054668	RT‐PCR	Forward	GAACACTTCGTCAACAAGGTCCC
			Reverse	GAGGAGAAAGCAACAACACCTTAATGA
		cDNA cloning	Forward	ATGTTCTCAGAGCTGCTCGGTACTCTGGTG
			Reverse	TTCAGTTGTTGTCTTGCTTGCTGCCCTGCT
*actb*	XM_003963431	RT‐PCR	Forward	AAGATGACCCAGATCATGTTTGAGA
			Reverse	CCTGAGTGTGTATGAGAAATGTGTG

### 2.3 Cloning of *Takifugu rubripes* TrAqps and expression in *Xenopus* oocytes

Full‐length cDNAs of TrAqps were isolated from the eye (TrAqp0a, 0b, 9a, 9b), kidney (TrAqp1aa, 1ab, 3a, 4a, 7, 8bb), intestine (TrAqp10aa, 10bb), swimbladder (TrAqp11a, 11b, 12), and brain (TrAqp14) of Japanese pufferfish by RT‐PCR using the primers designed based on the genomic database (Table S1) and a high‐fidelity DNA polymerase (KOD‐plus DNA polymerases, Toyobo). cDNAs were directly sequenced or subcloned into pGEMHE (Liman et al., 1992) and then sequenced. The sequences have been deposited under DDBJ/EMBL/GenBank accession numbers (TrAqp0a, LC735281, TrAqp0b, LC735282, TrAqp1aa, LC735283; TrAqp1ab, LC735284; TrAqp3a, LC735285; TrAqp4a, LC735286; TrAqp7, LC735287; TrAqp8bb, LC735288; TrAqp9a, LC735289; TrAqp9b, LC735290; TrAqp10aa, LC735291; TrAqp10bb, LC735292; TrAqp11a, LC735293; TrAqp11b, LC735294; TrAqp12, LC735295; TrAqp14, LC735296).

*Xenopus laevis* oocytes were dissociated with collagenase as described previously (Romero et al., 1998) and injected with 50 nL of water or a solution containing 0.5 ng/nL cRNA (25 ng/oocyte), using a Nanoject‐II injector (Drummond Scientific). Oocytes were incubated at 16°C in OR3 medium and studied for 3–5 days after injection. One liter of OR3 medium contained 0.7% w/v powdered Leibovitz L‐15 medium with l‐glutamine (Thermo Fisher Scientific), 50 mL of 10,000 U penicillin and 10,000 U streptomycin solution in 0.9% NaCl (Sigma‐Aldrich), and 5 mM HEPES (pH 7.50), and osmolarity was adjusted to 200 mosmol/kg with NaCl powder (Romero et al., 1998).

### 2.4 Swelling assay

The swelling of oocytes was monitored by measuring the diameter using a stereomicroscope (SZX9, Olympus) equipped with a charge‐coupled device (CCD) camera (DS‐Fi2, Nikon) every 30 s for 10 min. The volumes of oocytes were calculated assuming a spherical geometry. Oocytes incubated in ND96 (~200 mosmol/kg) were transferred to two‐times‐diluted ND96 (~100 mosmol/kg) for water transport assays (Takano et al., 2006). For glycerol, urea, or boric acid transport assays, oocytes were transferred to an isotonic solution containing ND96 supplemented with 180 mM glycerol, urea, or boric acid instead of NaCl, to adjust the osmolarity to ~200 mosmol/kg. Quantitative data of oocytes were obtained from at least three frogs. We performed the water, glycerol, urea, and boric acid transport assays described above using same batch of oocytes for each Aqp, and repeated the experiments using three or more frogs. To show time dependent increase in the cell volume in each solution, relative cell volume to the initial cell volume (*V*/*V*_*0*_) were calculated from the osmotic swelling data. Water permeability (*P*_water_) was calculated from the osmotic swelling data and the molar volume of water (*V*_*w*_ = 18 cm^3^/mol) as follows (Preston et al., 1992): *P*_water_ = [*V*_*o*_ × *d*(*V*/*V*_*o*_)/*dt*]/[*S* × *V*_*w*_ × (osm_in_ – osm_out_)], where *S*: initial oocyte surface area. Solute permeability (P_solute_) was calculated from the swelling data, the total osmolality of the system (osm_total_ = 200 mosM), and the solute gradient (sol_out_ – sol_in_) as follows (Carbrey et al., 2003): *P*_solute_ = osm_total_ × [*V*_*o*_ × *d*(*V*/*V*_*o*_)/*dt*]/[*S* × (sol_out_ – sol_in_)]. The statistical significance was evaluated by one‐way analysis of variance (ANOVA) followed by Dunnett's test using GraphPad Prism software (Version 5, GraphPad). Origin software (Version 8, OriginLab) was used to display the results in box plots.

The average values of *P*_water,_ *P*_glycerol_, *P*_urea_, and *P*_boric acid_ of oocytes expressing TrAqp0a, 1aa, 1ab, 3a, 4a, 7, 8bb, 9a, 9b, or 10bb and control oocytes were prepared from the swelling data. The correlation among the average values of *P*_water,_ *P*_glycerol_, *P*_urea_, and *P*_boric acid_ of TrAqp oocytes was calculated by Pearson's correlation using Excel software (Version 2019, Microsoft). Next, *P*_water_, *P*_glycerol_, *P*_urea_, and *P*_boric acid_ of oocytes expressing human Aqps (HsAqp1, 2, 3, 4, 5, 7, 8, 9, and 10) and control oocytes were similarly calculated from our previous swelling data (Ushio et al., 2022), and the average values were prepared as comparison. The correlation among the average values of *P*_water,_ *P*_glycerol_, *P*_urea_, and *P*_boric acid_ of TrAqp and HsAqp oocytes was again calculated by Pearson's correlation using Excel software.

### 2.5 Quantitative determination of boron concentration by inductively coupled plasma‐mass spectrometry (ICP‐MS)

Oocytes expressing TrAqps or water‐injected control oocytes were placed in ND96 containing 10 mM boric acid for 10 min at 23°C. Each oocyte was washed with ND96 for several seconds and dried after the removal of the washing solution. Dried *Xenopus* oocytes were digested with concentrated nitric acid in Teflon tubes, and the residues were dissolved in 0.08 M nitric acid containing 5 μg/L Be. Concentrations of boron‐10 and boron‐11 were measured by ICP‐MS (Agilent 7800 ICP‐MS, Agilent Technologies) using Be as an internal standard, and the sum of boron‐10 and boron‐11 concentrations is presented as the B concentration (Takano et al., 2006).

Quantitative data of oocytes from at least three frogs are presented as the mean ± SEM. Values for B content were compared among oocytes expressing TrAqps and control oocytes, and the statistical significances (*p* values) were calculated by one‐way analysis of variance (ANOVA) followed by Dunnett's test using GraphPad Prism software.

### 2.6 Ion‐selective microelectrode analysis

To measure the intracellular pH (pH_i_) of oocytes, H^+^ ion‐selective microelectrodes were prepared with a H^+^ ionophore I‐mixture B ion‐selective resin (Fluka Chemical) as described previously (Sciortino & Romero, 1999). pH_i_ was measured as the difference between the pH electrode and a KCl voltage electrode impaled into the oocyte using a two‐channel electrometer (FD223a, World Precision Instruments), and the membrane potential (V_m_) was measured as the difference between the KCl microelectrode and an extracellular calomel to a single electrometer (Electra 705, World Precision Instruments). pH electrodes were calibrated using pH 6.0 and 8.0 buffers (Wako Pure Chemical Industries), followed by a point calibration in ND96 (pH 7.5).

Oocytes were held on a nylon mesh in a chamber and perfused with a solution. V_m_ and pH_i_ were constantly monitored and recorded at 0.1 Hz using an analog‐to‐digital converter (PowerLab 8/35, AD Instruments) and LabChart software (AD Instruments). Solutions containing 1, 2, 5, and 10 mM boric acid were prepared by substituting NaCl with boric acid. The osmolarity and pH of all media were adjusted to ~200 mOsm and 7.5, respectively.

Quantitative data of oocytes from at least three frogs are presented as the mean ± SEM or in box plots using Origin software. Values for initial pH_i_, V_m_, ΔpH_i_/dt, and ΔV_m_ were compared between oocytes expressing TrAqps and control oocytes, and statistical significance (*p*‐value) was calculated by one‐way analysis of variance (ANOVA) followed by Dunnett's test using GraphPad Prism software.

## 3 RESULTS

### 3.1 Tissue distribution of *Takifugu rubripes aqp* genes

As previously reported by others (Chauvigne et al., 2019; Finn et al., 2014; Tingaud‐Sequeira et al., 2010; Yilmaz et al., 2020), database mining of the *Takifugu rubripes* genome sequence identified 16 *aqp* genes. Phylogenetic analysis among amino acid sequences of Aqps from *Takifugu*, human, zebrafish, and other related vertebrate animals confirmed that *Takifugu* has two paralogs for Aqp0 (TrAqp0a and TrAqp0b), two paralogs for Aqp1 (TrAqp1aa and TrAqp1ab), one ortholog for Aqp3 (TrAqp3a), one ortholog for Aqp4 (TrAqp4a), one ortholog for Aqp7 (TrAqp7), one ortholog for Aqp8 (TrAqp8bb), two paralogs for Aqp9 (TrAqp9a and TrAqp9b), two paralogs for Aqp10 (TrAqp10aa and TrAqp10bb), two paralogs for Aqp11 (TrAqp11a and TrAqp11b), one ortholog for Aqp12 (TrAqp12), and one ortholog for Aqp14 (TrAqp14) (Figure S1). As previously reported by others (Chauvigne et al., 2019; Finn et al., 2014; Tingaud‐Sequeira et al., 2010), no orthologs for *aqp2*, *aqp5*, and *aqp6* were identified in the *Takifugu* genome databases.

**FIGURE 1:**
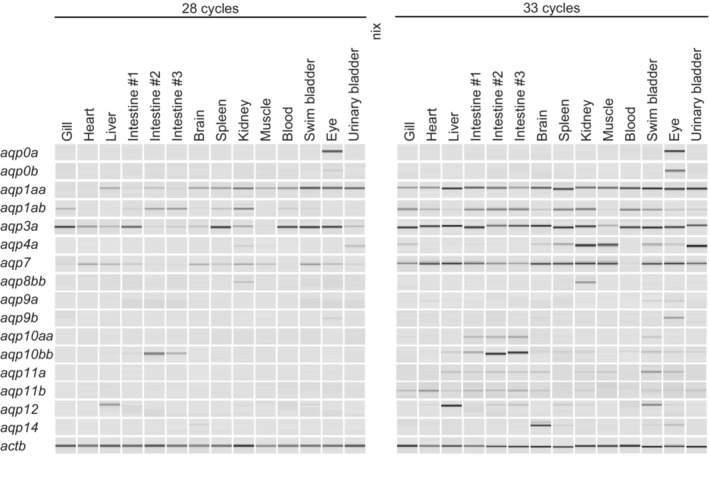
Tissue distribution of *aqp* family genes in the Japanese pufferfish *Takifugu rubripes*. Expression profiles of *aqp* in adult tissues were determined via semiquantitative RT‐PCR. Pseudo‐gel images of the PCR products were generated using a microchip electrophoresis system. *actb* (β‐Actin gene) was used as an internal control gene.

To determine the tissue distribution of TrAqps, RT‐PCR analyses (28 and 33 cycles) in 14 tissues of *Takifugu rubripes* were performed (Figure 1). The intestine and kidney are the sites of boric acid absorption and excretion, respectively (Kato et al., 2022). In *Takifugu rubripes*, TrAqp1aa, 1ab, 3a, 7, and 10bb were highly expressed in the intestine, and TrAqp1aa, 1ab, 3a, 4a, 7, and 8bb were highly expressed in the kidney.

### 3.2 Water permeability of *Xenopus* oocytes expressing TrAqps

**FIGURE 2:**
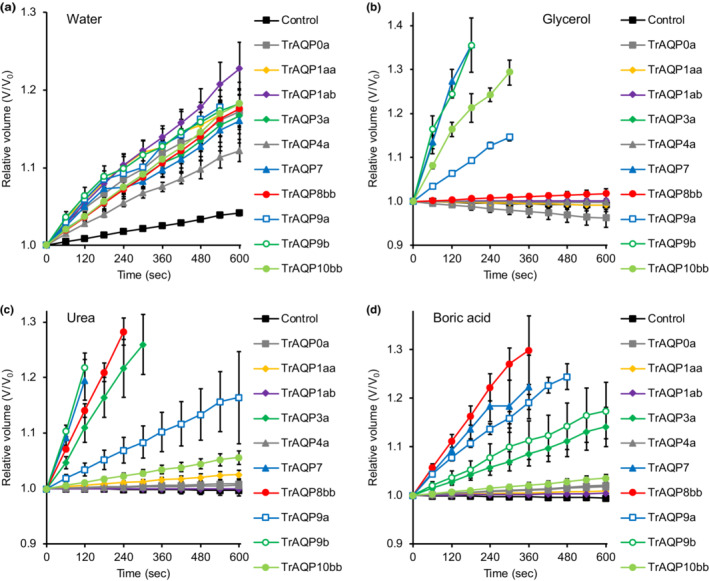
Increase in the volume of TrAqp‐expressing and control oocytes at the indicated timepoints (sec). Relative cell volume to the initial cell volume (*V*/*V*_*0*_) of oocytes in hypo‐osmotic solution (*a*), iso‐osmotic solution containing 180 mM glycerol (*b*), urea (*c*), or boric acid (*d*) was calculated from the swelling data. Values are shown as the mean ± SEM (*n* = 9–24).

**FIGURE 3:**
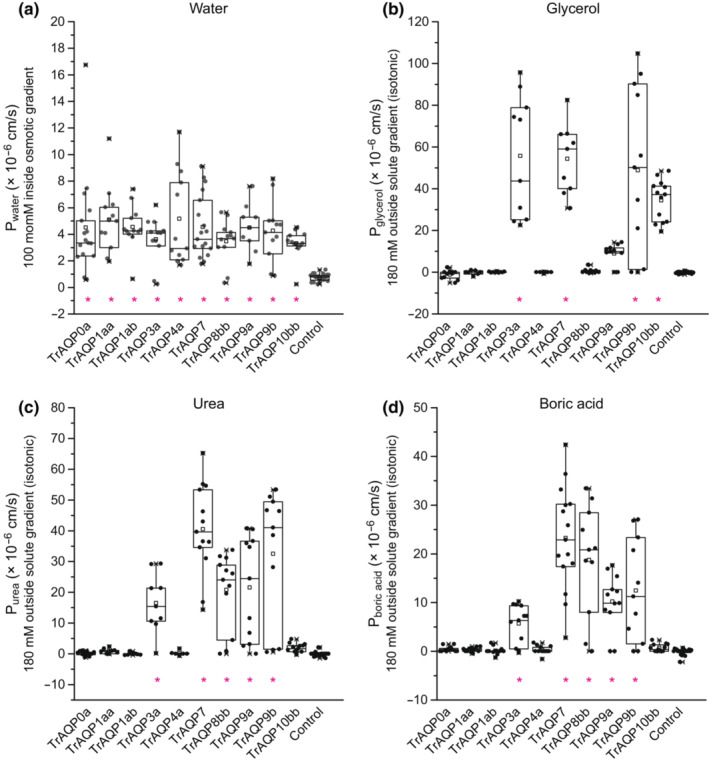
Water (*a*), glycerol (*b*), urea (*c*), and boric acid (*d*) permeability measurements of TrAqps by *Xenopus* oocyte swelling assay. P_water_, P_glycerol_, P_urea_, and P_boric acid_ values are presented as interquartile range from the 25–75 percentiles (box), range (whiskers), outliers (>1.5× interquartile range above upper quartile), mean (square in the box), and median (line in the box). Statistical significance was evaluated by one‐way ANOVA followed by Dunnett's test (*p < 0.05).

cRNAs for TrAqps were injected into *Xenopus* oocytes, and their water permeabilities were analyzed by the swelling assay. Oocytes expressing TrAqp0a, 1aa, 1ab, 3a, 4a, 7, 8bb, 9a, 9b, and 10bb showed significant volume gains of the cells (Figure 2a) and increase in P_water_ in the hypoosmotic solution (*n* = 16 for TrAqp0a, *p* < 0.05; *n* = 12 for TrAqp1aa, *p* < 0.05; *n* = 12 for TrAqp1ab, *p* < 0.05; *n* = 12 for TrAqp3a, *p* < 0.05; *n* = 12 for TrAqp4a, *p* < 0.05; *n* = 18 for TrAqp7, *p* < 0.05; *n* = 12 for TrAqp8bb, *p* < 0.05; *n* = 9 for TrAqp9a, *p* < 0.05; *n* = 12 for TrAqp9b, *p* < 0.05; *n* = 12 for TrAqp10bb, *p* < 0.05) (Figure 3a, Table 2), suggesting that these Aqps act as water channel in the plasma membrane of oocytes. In contrast, oocytes expressing TrAqp0b, TrAqp10aa, 11a, 11b, 12, and 14 showed weak or no increase in volume in the hypoosmotic solution (*n* = 12 for TrAqp0b; *n* = 16 for TrAqp10aa; *n* = 8 for TrAqp11a; *n* = 8 for TrAqp11b; *n* = 12 for TrAqp12; *n* = 12 for TrAqp14) compared to water‐injected negative control oocytes (*n* = 24) (data not shown).

**TABLE 2:** Water and solute permeability measurements of aquaporins in oocytes.

Protein	*P*_water_ (× 10^−6^ cm/s, 100 mosM inside osmotic gradient)	*P*_glycerol_ (× 10^−6^ cm/s, 180 mM outside solute gradient)	*P*_urea_ (× 10^−6^ cm/s, 180 mM outside solute gradient)	*P*_boric acid_ (× 10^−6^ cm/s, 180 mM outside solute gradient)
TrAqp0a	4.5 ± 3.8 (*n* = 16)	−1.1 ± 2.1 (*n* = 12)	0.1 ± 0.7 (*n* = 12)	0.4 ± 0.6 (*n* = 12)
TrAqp1aa	5.1 ± 2.5 (*n* = 12)	−0.2 ± 1 (*n* = 7)	0.9 ± 0.8 (*n* = 8)	0.3 ± 0.5 (*n* = 12)
TrAqp1ab	4.6 ± 1.7 (*n* = 12)	0.0 ± 0.2 (*n* = 9)	0.0 ± 0.4 (*n* = 9)	0.1 ± 0.8 (*n* = 12)
TrAqp3a	3.6 ± 1.7 (*n* = 12)	55.7 ± 29.2 (*n* = 10)	16.5 ± 9.6 (*n* = 9)	5.7 ± 4 (*n* = 12)
TrAqp4a	5.2 ± 3.5 (*n* = 12)	−0.12 ± 0.3 (*n* = 6)	0.22 ± 0.8 (*n* = 6)	0.3 ± 0.9 (*n* = 12)
TrAqp7	4.6 ± 2.3 (*n* = 18)	54.4 ± 16.8 (*n* = 9)	40.5 ± 14.8 (*n* = 13)	23.2 ± 10.7 (*n* = 15)
TrAqp8bb	3.5 ± 1.6 (*n* = 12)	0.5 ± 1.1 (*n* = 11)	20.8 ± 12.2 (*n* = 12)	18.8 ± 11.3 (*n* = 11)
TrAqp9a	4.5 ± 1.8 (*n* = 9)	8.9 ± 5 (*n* = 10)	21.5 ± 16.8 (*n* = 11)	10.2 ± 5.7 (*n* = 12)
TrAqp9b	4.3 ± 2.2 (*n* = 12)	48.9 ± 40.4 (*n* = 11)	32.5 ± 21.3 (*n* = 11)	12.5 ± 10.6 (*n* = 11)
TrAqp10bb	3.3 ± 1 (*n* = 12)	34.5 ± 9.8 (*n* = 13)	2.0 ± 1.5 (*n* = 12)	0.7 ± 0.8 (*n* = 12)
Control 1	0.7 ± 0.3 (*n* = 24)	−0.2 ± 0.4 (*n* = 24)	−0.1 ± 0.8 (*n* = 22)	−0.1 ± 0.6 (*n* = 24)
HsAqp1	3.7 ± 2.4 (*n* = 10)	−0.2 ± 0.8 (*n* = 12)	−0.1 ± 0.3 (*n* = 11)	−0.1 ± 0.6 (*n* = 16)
HsAqp2	4.7 ± 2.9 (*n* = 14)	−0.3 ± 0.3 (*n* = 12)	−0.1 ± 0.4 (*n* = 12)	0.1 ± 0.4 (*n* = 17)
HsAqp3	4.0 ± 1.9 (*n* = 23)	26.3 ± 7.1 (*n* = 13)	1.1 ± 1.2 (*n* = 18)	2.7 ± 2.2 (*n* = 20)
HsAqp4	3.1 ± 1.8 (*n* = 19)	−0.3 ± 0.4 (*n* = 15)	−0.3 ± 0.4 (*n* = 9)	0.3 ± 0.5 (*n* = 22)
HsAqp5	3.6 ± 2.6 (2 *n* = 2)	−0.4 ± 0.9 (*n* = 11)	−0.2 ± 0.3 (*n* = 12)	0.1 ± 0.9 (*n* = 18)
HsAqp7	2.2 ± 0.9 (*n* = 18)	10.0 ± 6.5 (*n* = 10)	0.6 ± 0.5 (*n* = 15)	3.0 ± 2.2 (*n* = 14)
HsAqp8	4.3 ± 2 (*n* = 16)	0.1 ± 0.6 (*n* = 12)	0.5 ± 0.5 (*n* = 12)	3.0 ± 1.4 (*n* = 19)
HsAqp9	3.0 ± 1 (*n* = 14)	22.0 ± 13.8 (*n* = 10)	11.0 ± 5.6 (*n* = 10)	28.2 ± 14.2 (*n* = 19)
HsAqp10	2.5 ± 0.8 (*n* = 17)	8.8 ± 3.3 (*n* = 12)	4.7 ± 1.8 (*n* = 11)	9.6 ± 3.4 (*n* = 19)
Control 2	0.7 ± 0.4 (*n* = 24)	0.0 ± 0.2 (*n* = 16)	0.0 ± 0.2 (*n* = 19)	0.2 ± 0.2 (*n* = 51)

Note: The values are mean ± standard deviation (*n*, total number of oocytes assayed). TrAqp, *Takifugu rubripes* aquaporin; HsAqp, *Homo sapiens* aquaporin. Results of HsAqps were recalculated from our previous study (Ushio et al., 2022). Control 1 and 2 indicate results of water‐injected oocytes in this and the previous study, respectively.

### 3.3 Glycerol and urea permeability of *Xenopus* oocytes expressing TrAqps

Next, we analyzed the glycerol and urea permeabilities of oocytes expressing TrAqps by the swelling assay using iso‐osmotic solution containing 180 mM glycerol or urea. In the iso‐osmotic solution containing glycerol, oocytes expressing TrAqp3a, 7, 9b, and 10bb showed a significant increase in the cell volume (Figure 2b) and P_glycerol_ (*n* = 10 for TrAqp3a, *p* < 0.05; *n* = 9 for TrAqp7, *p* < 0.05; *n* = 11 for TrAqp9b, *p* < 0.05; *n* = 13 for TrAqp10bb, *p* < 0.05; *n* = 24 for control) (Figure 3b, Table 2), suggesting that these Aqps acted as glycerol channels.

In the iso‐osmotic solution containing urea, oocytes expressing TrAqp3a, 7, 8bb, 9a, and 9b showed a significant increase in the cell volume (Figure 2c) and P_urea_ (*n* = 9 for TrAqp3a, *p* < 0.05; *n* = 13 for TrAqp7, *p* < 0.05; *n* = 12 for TrAqp8bb, *p* < 0.05; *n* = 11 for TrAqp9a, *p* < 0.05; *n* = 11 for TrAqp9b, *p* < 0.05; *n* = 22 for control) (Figure 3c, Table 2). These results suggest that the above Aqps act as urea channels.

### 3.4 Boric acid permeability of *Xenopus* oocytes expressing TrAqps

The boric acid permeability of oocytes expressing TrAqps was analyzed using the swelling assay with an iso‐osmotic solution containing 180 mM boric acid. In the iso‐osmotic solution containing boric acid, oocytes expressing TrAqp3a, 7, 8bb, 9a, and 9b showed a significant increase in cell volume (Figure 2d) and P_boric acid_ (*n* = 12 for TrAqp3a, *p* < 0.05; *n* = 15 for TrAqp7, *p* < 0.05; *n* = 11 for TrAqp8bb, *p* < 0.05; *n* = 12 for TrAqp9a, *p* < 0.05; *n* = 11 for TrAqp9b, *p* < 0.05; *n* = 24 for control) (Figure 3d, Table 2).

**FIGURE 4:**
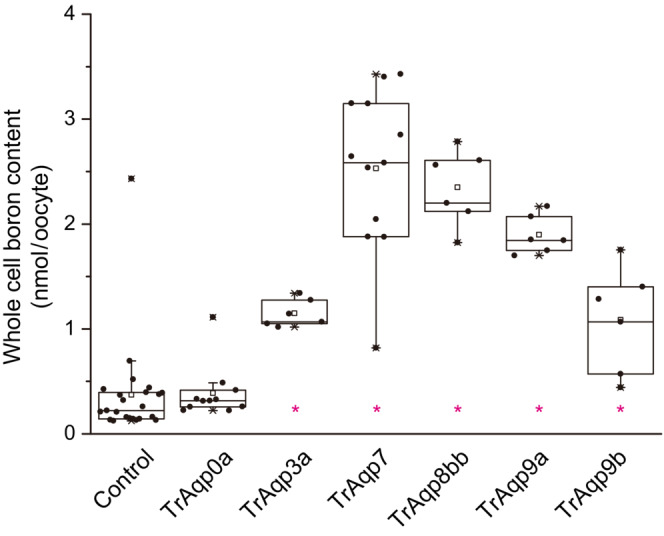
Boric acid uptake activity of TrAqp oocytes measured as whole‐cell boron content using inductively coupled plasma‐mass spectrometry. Values are presented as interquartile range from the 25–75 percentiles (box), range (whiskers), outliers (>1.5× interquartile range above upper quartile), mean (square in the box), and median (line in the box). Statistical significance was evaluated by one‐way ANOVA followed by Dunnett's test (*p < 0.05, *n* = 6–24).

To directly confirm that exogenous expression of TrAqp3a, 7, 8bb, 9a, and 9b mediated boric acid influx, we next analyzed the whole‐cell boron content of oocytes expressing TrAqps immersed in a solution containing boric acid and compared it with that of water‐injected control oocytes. After exposure to an iso‐osmotic solution containing 10 mM boric acid for 10 min, the whole‐cell boron content of these oocytes was significantly increased (*n* = 6 for TrAqp3a, *p* < 0.05; *n* = 12 for TrAqp7, *p* < 0.05; *n* = 6 for TrAqp8bb, *p* < 0.05; *n* = 6 for TrAqp9a, *p* < 0.05; *n* = 6 for TrAqp9b, *p* < 0.05; *n* = 24 for control) (Figure 4). In contrast, the whole‐cell boron content of oocytes expressing TrAqp0a (*n* = 6), an orthodox Aqp, was similar to that of control oocytes.

**FIGURE 5:**
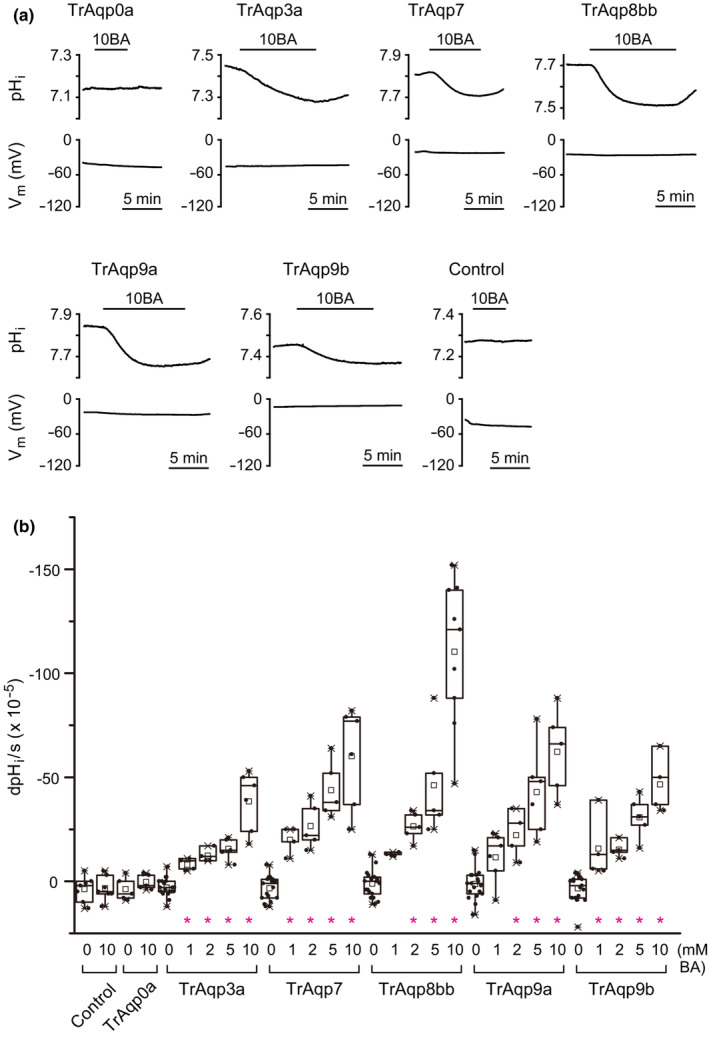
B(OH)_3_ permeability of oocytes expressing TrAqp3a, 7, 8bb, 9a, and 9b. *A*: Representative traces of changes in the intracellular pH (pH_i_) and membrane potential (V_m_) of an oocyte expressing TrAqp0a, 3a, 7, 8bb, 9a, or 9b, or a control oocyte. 10BA, 10 mM boric acid. *B*: The summary of pH changes (dpH_i_/s) in oocytes expressing Aqps and control in solution containing 0, 1, 2, 5, or 10 mM boric acid. BA, boric acid. Values are presented as interquartile range from the 25–75 percentiles (box), range (whiskers), outliers (>1.5× interquartile range above upper quartile), mean (square in the box), and median (line in the box). Statistical significance was evaluated by one‐way ANOVA followed by Dunnett's test (*p < 0.05, *n* = 4–25).

Boric acid (B(OH)_3_) is in equilibrium with borate (B(OH)_4_^−^) in aqueous solutions. To determine which form is transported in oocytes expressing TrAqps, B(OH)_3_ or B(OH)_4_^−^, we analyzed changes in intracellular pH (pH_i_) of oocytes expressing TrAqps in a solution containing boric acid because B(OH)_3_ influx elicits intracellular acidification, but the influx of B(OH)_4_^−^ elicits intracellular alkalization and membrane hyperpolarization (Kato et al., 2022; Ushio et al., 2022). Changes in pH_i_ were analyzed in oocytes expressing TrAqp3a, 7, 8bb, 9a, and 9b in an iso‐osmotic solution containing boric acid. Water‐injected oocytes and oocytes expressing TrAqp0a were used as negative controls. In oocytes expressing TrAqp3a, 7, 8bb, 9a, and 9b, exposure to the iso‐osmotic solution containing 10 mM boric acid elicited a significant decrease in pH_i_ (*n* = 6 for TrAqp3a, *p* < 0.05; *n* = 6 for TrAqp7, *p* < 0.05; *n* = 11 for TrAqp8bb, *p* < 0.05; *n* = 5 for TrAqp9a, *p* < 0.05; *n* = 4 for TrAqp9b, *p* < 0.05); however, there was no change in the membrane potential (Figure 5a). No changes in pH_i_ were observed in water‐injected oocytes or TrAqp0a oocytes (*n* = 5 for TrAqp0a; *n* = 7 for control). Changes in pH_i_ were dependent on the boric acid concentrations of the bath solutions (Figure 5b). These results suggest that TrAqp3a, 7, 8bb, 9a, and 9b transport B(OH)_3_ and act as boric acid channels.

### 3.5 Relationships among water, glycerol, urea, and boric acid permeability of *Xenopus* oocytes expressing TrAqps

**FIGURE 6:**
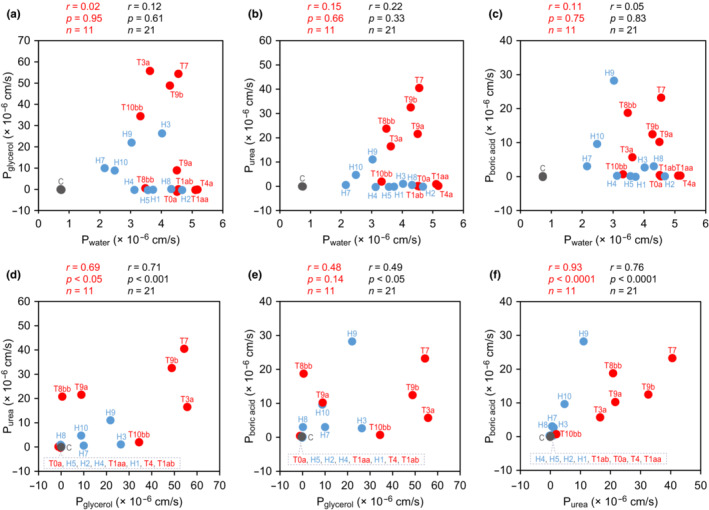
Scatter plots and Pearson's correlation coefficient (*r*) among water, glycerol, urea, and boric acid permeabilities of oocytes expressing pufferfish TrAqps. (*A*‐*F*) The average values of water and solute permeabilities (*P*_water_, *P*_glycerol_, *P*_urea_, and *P*_boric acid_, shown in Table 2) expressing pufferfish TrAqps are plotted in red, and those of water‐injected oocytes were plotted in gray. The average values of water and solute permeabilities of oocytes expressing human HsAqps were recalculated from our previous study (Ushio et al., 2022) (Table 2) were plotted as comparison in blue. The correlations (*r*) calculated from the average values of TrAqp and control (*n* = 11) were shown with *p* value in red, and those calculated from the average values of TrAqp, HsAqp, and each control (*n* = 21) were shown with *p* value in black. T0a, TrAqp0a; T1aa, TrAqp1aa; T1ab, TrAqp1ab; T3a, TrAqp3a; T4a, TrAqp4a; T7, TrAqp7; T8bb, TrAqp8bb; T9a, TrAqp9a; T9b, TrAqp9b; T10bb, TrAqp10bb; H1, HsAqp1; H2, HsAqp2; H3, HsAqp3; H4, HsAqp4; H5, HsAqp5; H7, HsAqp7; H8, HsAqp8; H9, HsAqp9; H10, HsAqp10; C, control.

Water permeability (P_water_) and solute permeabilities (P_glycerol_, P_urea_, and P_boric acid_) of oocytes expressing TrAqp0a, 1aa, 1ab, 3a, 4a, 7, 8bb, 9a, 9b, or 10bb and control oocytes were summarized in Table 2 and plotted in Figure 6, and a total of 11 plots were used (i.e., *n* = 11) to analyze the Pearson's correlation. P_water_ of TrAqp oocytes was not correlated with P_glycerol_, P_urea_, and P_boric acid_ of TrAqp oocytes (*r* = 0.02–0.15, *n* = 11, Figure 6 **, a–c**). The P_glycerol_ was weakly correlated with the P_urea_ (*r* = 0.69, *n* = 11, *p* < 0.05, Figure 6d) but not with the P_boric acid_ (*r* = 0.48, *n* = 11, *p* = 0.14, Figure 6e). The P_boric acid_ was correlated with the P_urea_ (*r* = 0.93, *n* = 11, *p* < 0.0001, Figure 6f).

As comparison, P_water_, P_glycerol_, P_urea_, and P_boric acid_ values of oocytes expressing human Aqps (HsAqps) from our previous data (Ushio et al., 2022) were also calculated (Table 2) and plotted in Figure 6. P_water_ of TrAqp and HsAqp oocytes was not correlated with that of P_glycerol_, P_urea_, and P_boric acid_ (*r* = 0.05–0.22, *n* = 21, Figure 6 **, a–c**). P_glycerol_ was correlated with P_urea_ (*r* = 0.71, *n* = 21, *p* < 0.001, Figure 6d) and was weakly correlated with P_boric acid_ (*r* = 0.49, *n* = 21, *p* < 0.05, Figure 6e). P_boric acid_ was correlated with P_urea_ (*r* = 0.76, *n* = 21, *p* < 0.0001, Figure 6f).

## 4 DISCUSSION

The number of *aqp* genes in the genome database of *Takifugu rubripes*, a marine teleost, is 16, which is smaller than that of the zebrafish *Danio rerio*, which has 18 *aqp* genes (Tingaud‐Sequeira et al., 2010). These numbers are much smaller than those of the common carp (*Cyprinus carpio*), which has a tetraploidized genome with 37 *aqp* genes (Dong et al., 2016). Neither *Takifugu* nor zebrafish have *aqp2*, *aqp5*, and *aqp6* genes. In the genomes of tetrapods, these three *aqp* genes are tandemly located within the same chromosome (Ma et al., 1997). As previously discussed by other research groups (Finn et al., 2014), functional ancestral genes encoding Aqp2/5/6 may have been lost from the genomes of the common ancestral species of ray‐finned (Actinopterygii) fishes, and *aqp2*, *aqp5*, and *aqp6* may have evolved in the ancestral tetrapod species by tandem gene duplication. The number of paralog genes for *aqp3* and *aqp8* are different between *Takifugu* and zebrafish, possibly due to lineage‐specific gain and loss of the genes (Cao & Shi, 2019).

To characterize TrAqp functions, we used the *Xenopus* oocyte expression system (Ushio et al., 2022) and first analyzed whether they transport water, glycerol, and urea, which are known substrates of the Aqp family. Results of the oocyte swelling assay showed that TrAqp0a, TrAqp1aa, TrAqp1ab, and TrAqp4a are water‐specific aquaporins, which are similar to their orthologs in mammals (Borgnia et al., 1999) and zebrafish (Tingaud‐Sequeira et al., 2010), suggesting that Aqp0, 1, and 4 are evolutionarily conserved water‐specific aquaporins. TrAqp3a, 7, and 9b transport water, glycerol, and urea, and these activities are similar to those of zebrafish orthologs (Tingaud‐Sequeira et al., 2010), showing that these Aqps have the widest range of substrate selectivity. Interestingly, TrAqp8bb and 9a are water and urea transporters, and this substrate selectivity is similar to that of zebrafish DrAqp8aa and 8ab, but not DrAqp9a, because zebrafish DrAqp9a transports water, glycerol, and urea. These results suggest that teleost Aqp8 is an evolutionarily conserved water and urea channel. TrAqp10bb acts as a water and glycerol transporter with no urea transport activity. The urea transport activity of zebrafish DrAqp10bb is much weaker than its urea transport activity, whereas zebrafish DrAqp10aa (Tingaud‐Sequeira et al., 2010) and human HsAqp10 (Ishibashi et al., 2002; Ushio et al., 2022) transport glycerol and urea efficiently, suggesting that teleost Aqp10bb is an evolutionarily conserved water and glycerol transporter.

The present experiments did not show significant activity of TrAqp0b, TrAqp10aa, 11a, 11b, 12, and 14. In mammals, Aqp11 and 12 are known to be intracellularly localized (Gorelick et al., 2006; Itoh et al., 2005). Therefore, the activities of intracellular TrAqp11a, 11b, and 12 may not be measured by the oocyte swelling assay probably due to their intracellular localizations. In zebrafish, activities of DrAqp0b, 10aa, and 14 were measured by others using *Xenopus* oocyte expression system (Chauvigne et al., 2019; Tingaud‐Sequeira et al., 2010). However, we could not detect activities of their pufferfish orthologs, TrAqp0b, TrAqp10aa, and 14 in this study by unknown reason, probably due to a failure of protein synthesis or trafficking in *Xenopus* oocyte expression system. Further analysis is required to know the activity of TrAqp0b, TrAqp10aa, and 14.

We next tested whether TrAqps transports boric acid as a new substrate for fish Aqps. By using multiple techniques such as swelling assay, element quantification, and pH_i_ measurement, the expression of five Aqps, TrAqp3a, 7, 8bb, 9a, and 9b, increased the cell membrane permeability of B(OH)_3_. This is the first demonstration of fish Aqp acting as a boric acid transport system, probably as a channel. This activity is advantageous for the passive diffusion of boric acid, and these Aqps may have important roles in supplying boric acid to various cells and maintaining the distribution of boric acid in the body of marine teleosts. The results also indicated that all water‐specific TrAqps (TrAqp0a, 0b, 1aa, 1ab, and 4a) and the aquaglyceroporin TrAqp10bb could not transport boric acid, because the expression of these TrAqps did not elicit an increase in the cell volume of oocytes in the iso‐osmotic solution containing boric acid.

We recently showed that pufferfishes acclimated to SW absorb boric acid in the intestine and excrete it through the kidney (Kato et al., 2022). Among the six boric acid permeable TrAqps shown in the present study, TrAqp3a and 7 were expressed in the intestine, and TrAqp3a, 7, and 8bb were expressed in the kidney, suggesting that these TrAqps may have potential roles in intestinal and renal handling of boric acid in pufferfish. However, further analysis is required to understand the molecular mechanisms by which these Aqps work in the intestine and kidney of marine teleosts.

Recently, we also showed that human HsAqp3, 7, 8, 9, and 10 act as boric acid transport systems when expressed in *Xenopus* oocytes (Ushio et al., 2022). Therefore, the boric acid transport activity may be an evolutionarily conserved characteristic of Aqp3, 7, 8, and 9. We observed a striking difference in activity between HsAqp10 and TrAqp10bb, that is, human HsAqp10 transports water, glycerol, urea, and boric acid, whereas TrAqp10bb transports water and glycerol, but not urea or boric acid. The difference in the activity of Aqp10 among species raises a new question as to which activity of Aqp10 is ancestral and when and how the substrate specificity of Aqp10 changes during the evolution of vertebrates.

## AUTHOR CONTRIBUTIONS

S.K., E.W., N.H., Y.K., S.H., M.F.R., and A.K. conceived and designed the study; S.K., E.W., N.H., Y.K., T.K., A.N., T.U., G.I., K.M., K.K., and A.K. performed experiments; S.K., E.W., N.H., Y.K., T.K., A.N., K.U., J.K., K.K., and A.K. analyzed the data; T.K., S.H., T.F., M.F.R., and A.K. supervised the experiments; S.K., E.W., A.N., K.U., J.K., and A.K. prepared the figures; S.K., E.W., M.F.R., and A.K. drafted the manuscript; M.F.R. and A.K. edited the manuscript; S.K., E.W., T.K., S.H., T.F., M.F.R., and A.K. approved the final version of the manuscript.

## FUNDING INFORMATION

This work was supported by JSPS KAKENHI (Grant Numbers 22,370,029, 25,650,114, 26,292,113, 17H03870, 19H05637, and 21H02281). This work in the Romero lab was supported by the NIH (DK092408; DK100227) and the Mayo Foundation.

## CONFLICT OF INTEREST STATEMENT

No conflicts of interest, financial or otherwise, are declared by the authors. KU is an employee of Chugai Pharmaceutical Company Limited. The funder did not have any role in the study design, data collection and analysis, decision to publish, or preparation of the manuscript.

## DATA DEPOSITION

The sequences reported in this paper have been deposited in the GenBank database (accession no. LC735281 ‐ LC735296).

## ETHICS STATEMENT

All *Xenopus* care and oocyte harvest protocols were conducted in accordance with the National Institutes of Health “Guide for the Care and Use of Laboratory Animals.” Frogs were housed and cared for following the approval of the Institutional Animal Care and Use Committee of the Mayo Clinic College of Medicine and in accordance with a manual approved by the Institutional Animal Experiment Committee of the Tokyo Institute of Technology.

## Supporting information


**Supplementary Fig. S1.** Phylogenetic analyses of *Takifugu rubripes* Aqp family. A phylogenetic tree of Aqp families was constructed using the maximum‐likelihood method with ClustalW and MEGA4. Numbers indicate bootstrap values. List of amino acid sequences used for the analysis is shown in Table S1.

**Supplementary Table S1.** List of amino acid sequences used for the phylogenetic analysis.
