# Supplementary material for: Boric acid transport activity of marine teleost aquaporins expressed in Xenopus oocytes
Source: Physiol Rep. 2023 Mar 26;11(6):e15655. doi: 10.14814/phy2.15655 (PMC10040401; doi:10.14814/phy2.15655)
Supplement: Supplementary file 2 — Supplementary Table S1. List of amino acid sequences used for the phylogenetic analysis. [file PHY2-11-e15655-s001.pdf]

Supplementary Table S1. List of amino acid sequences used for the phylogenetic analysis.

No.	Common name	Scientific name	Protein name	Accession number
1	Japanese pufferfish	*Takifugu rubripes*	Aqp0a	LC735281, XM_003963091, ENSTRUT00000009841
2	Japanese pufferfish	*Takifugu rubripes*	Aqp0b	LC735282, XM_003963426, ENSTRUT00000040237
3	Japanese pufferfish	*Takifugu rubripes*	Aqp1aa	LC735283, XM_003975326, ENSTRUT00000083310
4	Japanese pufferfish	*Takifugu rubripes*	Aqp1ab	LC735284, XM_003975371, ENSTRUT00000034800.3
5	Japanese pufferfish	*Takifugu rubripes*	Aqp3a	LC735285, XM_003975233
6	Japanese pufferfish	*Takifugu rubripes*	Aqp4a	LC735286, XM_011618835, ENSTRUT00000076888
7	Japanese pufferfish	*Takifugu rubripes*	Aqp7	LC735287, XM_003973563, ENSTRUT00000039663
8	Japanese pufferfish	*Takifugu rubripes*	Aqp8bb	LC735288, XM_003964545, ENSTRUT00000007374
9	Japanese pufferfish	*Takifugu rubripes*	Aqp9a	LC735289, XM_003967709, ENSTRUT00000021115
10	Japanese pufferfish	*Takifugu rubripes*	Aqp9b	LC735290, XM_003969979, ENSTRUT00000045810
11	Japanese pufferfish	*Takifugu rubripes*	Aqp10aa	LC735291, XM_011605829, ENSTRUT00000066901
12	Japanese pufferfish	*Takifugu rubripes*	Aqp10bb	LC735292, XM_003969282, ENSTRUT00000024481
13	Japanese pufferfish	*Takifugu rubripes*	Aqp11a	LC735293, XM_003968221, ENSTRUT00000017753
14	Japanese pufferfish	*Takifugu rubripes*	Aqp11b	LC735294, XM_003970961, ENSTRUT00000050959
15	Japanese pufferfish	*Takifugu rubripes*	Aqp12	LC735295, XM_003973857, ENSTRUT00000041698
16	Japanese pufferfish	*Takifugu rubripes*	Aqp14	LC735296, XM_003963431, ENSTRUT00000054668
17	spotted green pufferfish	*Tetraodon nigroviridis*	Aqp0a	CAAE01014991, ENSTNIT00000005037
18	spotted green pufferfish	*Tetraodon nigroviridis*	Aqp0b	CAAE01014729, ENSTNIT00000015776
19	spotted green pufferfish	*Tetraodon nigroviridis*	Aqp1aa	CAAE01014556, ENSTNIT00000011493
20	spotted green pufferfish	*Tetraodon nigroviridis*	Aqp1ab	ENSTNIT00000001619
21	spotted green pufferfish	*Tetraodon nigroviridis*	Aqp3a	CAAE01008553, ENSTNIT00000006577
22	spotted green pufferfish	*Tetraodon nigroviridis*	Aqp4a	CAG07606, ENSTNIT00000018500
23	spotted green pufferfish	*Tetraodon nigroviridis*	Aqp7	CAG01413, ENSTNIT00000013786
24	spotted green pufferfish	*Tetraodon nigroviridis*	Aqp8bb	CAAE01015037, ENSTNIT00000021394
25	spotted green pufferfish	*Tetraodon nigroviridis*	Aqp9a	CAAE01014769, ENSTNIT00000016917
26	spotted green pufferfish	*Tetraodon nigroviridis*	Aqp9b	CAAE01014581, ENSTNIT00000012389
27	spotted green pufferfish	*Tetraodon nigroviridis*	Aqp10aa	CAAE01015044, ENSTNIT00000021743
28	spotted green pufferfish	*Tetraodon nigroviridis*	Aqp10bb	CAAE01015022, ENSTNIT00000020947
29	spotted green pufferfish	*Tetraodon nigroviridis*	Aqp12	CAAE01015025, ENSTNIT00000021006
30	spotted green pufferfish	*Tetraodon nigroviridis*	Aqp14	MN168322, ENSTNIT00000015775
31	zebrafish	*Danio rerio*	Aqp0a	FJ666326, ENSDART00000054238

32	zebrafish	*Danio rerio*	Aqp0b	FJ655389, ENSDART00000023793
33	zebrafish	*Danio rerio*	Aqp1aa	DQ887675, ENSDART00000032459
34	zebrafish	*Danio rerio*	Aqp1ab	NM_001135682, ENSDART00000127523
35	zebrafish	*Danio rerio*	Aqp3a	NM_213468, ENSDART00000009182
36	zebrafish	*Danio rerio*	Aqp3b	NM_001166121, ENSDART00000101241
37	zebrafish	*Danio rerio*	Aqp4b	FJ666327, ENSDART00000011287
38	zebrafish	*Danio rerio*	Aqp7	NM_199910, ENSDART00000123522
39	zebrafish	*Danio rerio*	Aqp8aa	FJ655386, ENSDART00000066382
40	zebrafish	*Danio rerio*	Aqp8ab	EU341834, ENSDART00000105952
41	zebrafish	*Danio rerio*	Aqp8bb	FJ695516, ENSDART00000122968
42	zebrafish	*Danio rerio*	Aqp9a	NM_001033096
43	zebrafish	*Danio rerio*	Aqp9b	NM_001177744, ENSDART00000075513
44	zebrafish	*Danio rerio*	Aqp10a	NM_001002349, ENSDART00000124718
45	zebrafish	*Danio rerio*	Aqp10b	XM_005159392, ENSDART00000140719
46	zebrafish	*Danio rerio*	Aqp11	NM_001327893, ENSDART00000165002
47	zebrafish	*Danio rerio*	Aqp12	NM_001045862, ENSDART00000063533
48	zebrafish	*Danio rerio*	Aqp14	XM_005174125, ENSDART00000162405
49	western clawed frog	*Xenopus tropicalis*	Aqp0	NM_001097347, ENSXETT00000047158
50	western clawed frog	*Xenopus tropicalis*	Aqp1	NM_001005829, ENSXETT00000111653
51	western clawed frog	*Xenopus tropicalis*	Aqp2	NM_001015749, ENSXETT00000043976
52	western clawed frog	*Xenopus tropicalis*	Aqp3	NM_001016845, ENSXETT00000004583
53	western clawed frog	*Xenopus tropicalis*	Aqp4	NM_001317845, ENSXETT00000060637
54	western clawed frog	*Xenopus tropicalis*	Aqp5	NM_001310112, ENSXETT00000043991
55	western clawed frog	*Xenopus tropicalis*	Aqp7	NM_001015726, ENSXETT00000061610
56	western clawed frog	*Xenopus tropicalis*	Aqp8	NM_001114256, ENSXETT00000062511
57	western clawed frog	*Xenopus tropicalis*	Aqp9	XM_002937673, ENSXETT00000023755
58	western clawed frog	*Xenopus tropicalis*	Aqp10	XM_002943358, ENSXETT00000049658
59	western clawed frog	*Xenopus tropicalis*	Aqp11	XM_004912199, ENSXETP00000058403
60	western clawed frog	*Xenopus tropicalis*	Aqp12	XM_031902958, ENSXETT00000119773
61	western clawed frog	*Xenopus tropicalis*	Aqp13	XM_002940465, ENSXETT00000035583.5
62	western clawed frog	*Xenopus tropicalis*	Aqp14	AAMC04000002, ENSXETT00000061170
63	mouse	*Mus musculus*	Aqp0	NM_008600, ENSMUST00000026455
64	mouse	*Mus musculus*	Aqp1	NM_007472, ENSMUST00000004774

65	mouse	*Mus musculus*	Aqp2	NM_009699, ENSMUST00000023752
66	mouse	*Mus musculus*	Aqp3	NM_016689, ENSMUST00000055327
67	mouse	*Mus musculus*	Aqp4	NM_009700, ENSMUST00000079081
68	mouse	*Mus musculus*	Aqp5	NM_009701, ENSMUST00000088200
69	mouse	*Mus musculus*	Aqp6	NM_175087, ENSMUST00000023754
70	mouse	*Mus musculus*	Aqp7	NM_001378639, ENSMUST00000030136
71	mouse	*Mus musculus*	Aqp8	NM_007474, ENSMUST00000098056
72	mouse	*Mus musculus*	Aqp9	NM_022026, ENSMUST00000074465
73	mouse	*Mus musculus*	Aqp11	NM_175105, ENSMUST00000206389
74	mouse	*Mus musculus*	Aqp12	NM_177587, ENSMUST00000059676
75	human	*Homo sapiens*	Aqp0	NM_012064, ENST00000257979
76	human	*Homo sapiens*	Aqp1	NM_198098, ENST00000441328
77	human	*Homo sapiens*	Aqp2	NM_000486, ENST00000199280
78	human	*Homo sapiens*	Aqp3	NM_004925, ENST00000297991
79	human	*Homo sapiens*	Aqp4	NM_001650, ENST00000383168
80	human	*Homo sapiens*	Aqp5	NM_001651, ENST00000293599
81	human	*Homo sapiens*	Aqp6	NM_001652, ENST00000315520
82	human	*Homo sapiens*	Aqp7	NM_001376192, ENST00000377425
83	human	*Homo sapiens*	Aqp8	NM_001169, ENST00000219660
84	human	*Homo sapiens*	Aqp9	NM_020980, ENST00000219919
85	human	*Homo sapiens*	Aqp10	NM_080429, ENST00000324978
86	human	*Homo sapiens*	Aqp11	NM_173039, ENST00000313578
87	human	*Homo sapiens*	Aqp12	NM_001102467, ENST00000429564
